# Tardigrade Survival Limits in High-Speed Impacts—Implications for Panspermia and Collection of Samples from Plumes Emitted by Ice Worlds

**DOI:** 10.1089/ast.2020.2405

**Published:** 2021-07-06

**Authors:** Alejandra Traspas, Mark J. Burchell

**Affiliations:** Centre for Astrophysics and Planetary Science, School of Physical Sciences, University of Kent, Canterbury, UK.

**Keywords:** Panspermia, Europa, Enceladus, Hypervelocity impact processes

## Abstract

The ability of tardigrades to survive impact shocks in the kilometer per second and gigapascal range was investigated. When rocks impact planetary surfaces, the impact speeds and shock pressures are in the kilometer per second and gigapascal range. This investigation tested whether tardigrades can survive in impacts typical of those that occur naturally in the Solar System. We found that they can survive impacts up to 0.9 km s^−1^, which is equivalent to 1.14 GPa shock pressure, but cannot survive impacts above this. This is significantly less than the static pressure limit and has implications for tardigrade survival in panspermia models. The potential survival of tardigrades in impacts of terrestrial impact ejecta on the Moon is shown to be impossible for the average lunar impact speed of such ejecta. However, a notable fraction (around 40%) of such ejecta impact at vertical speeds low enough to permit survival. Similarly, martian impact ejecta striking Phobos, for example, at a typical impact speed will not permit viable transfer of tardigrade-like organisms, but if a fraction of such material had a lower impact speed, survival may be possible. We also consider the implications of this for the collection of viable samples by spacecraft transiting the plumes of icy water worlds such as Europa and Enceladus. We have found the limit on survival of shocks to be around 1 GPa, which is instrumental in determining appropriate mission scenarios and collection methods for the acquisition of viable materials.

## 1. Introduction

**FIG. 1.**
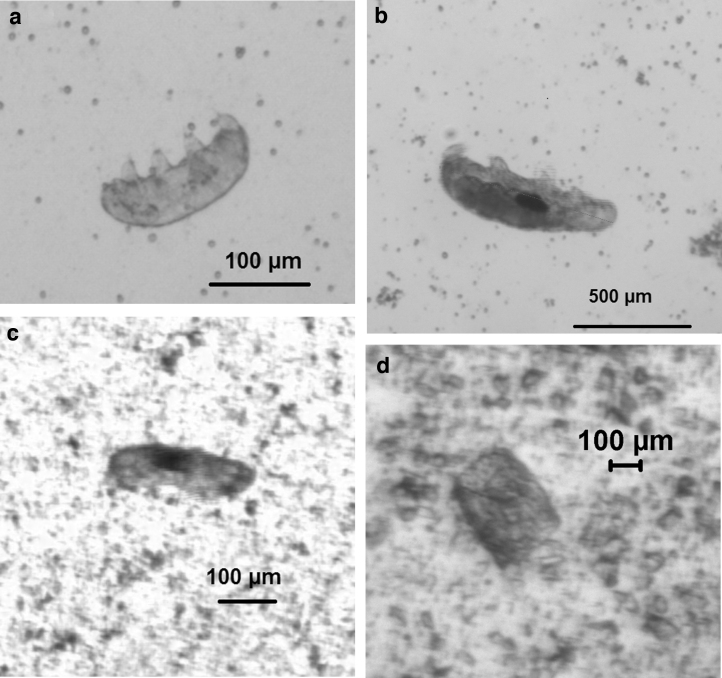
(**a**, **b**) Example tardigrades before impact testing. Tardigrades ranged in size from 150 to 850 μm. (**c**) Tardigrade recovered after an impact at 0.728 km s^−1^. (**d**) Tardigrade fragment from shot at 0.901 km s^−1^.

Tardigrades are remarkable diminutive creatures of anywhere from 100 to 1000 microns in maximum length and live mainly in freshwater environments (Weronika and Lukasz, 2017). Examples are shown in Fig. 1a and 1b. Tardigrades are also able to survive extreme environmental conditions such as low temperatures, vacuum, and radiation (Persson *et al*., 2011; Schill and Hengherr, 2018; Jönsson, 2019) and have survived exposure in space on the exterior of space vehicles (Jönsson *et al*., 2008; Rebecchi *et al*., 2009; Persson *et al*., 2011). This ability to survive extreme conditions has led to suggestions that they could be a vector for panspermia, that is, natural movement of life between bodies in space (*e.g.,* see Veras *et al*., 2018, or Burchell, 2004, for a review of panspermia). Particular models of panspermia, such as lithopanspermia (Melosh, 1988), involve movement of rocks that contain life from one planetary surface to another, that is, launch into space from the surface of a planet on impact ejecta and subsequent arrival on a new body at high speed. Both ejection and arrival involve accelerations and shocks.

While rocks from other bodies such as Mars are known to arrive on Earth, their measured transit times in space mean the integrated radiation dose even at their core will have killed any known terrestrial organisms (Clark, 2001). In theory, shorter transit times are possible, so this does not *a priori* rule out successful panspermia. However, the magnitude of the shock processes involved in lithopanspermia is more of a limiting factor. The final step alone, impact on a new home, typically involves an impact at speeds measured in kilometers per second, and the resulting shock pressures are in the tens of gigapascals range and upward. Past research has shown that microbes and spores can survive impacts at speeds of up to around 5 km s^−1^ (Burchell *et al.*, 2000) and peak shock pressures of around 40 GPa or more (Burchell *et al.*, 2001; Horneck *et al*., 2001). However, survival rates are low, measured in 1 per 10^4^ or 10^6^ (see, *e.g.,* Burchell *et al.*, 2004; Horneck *et al*., 2008; Price *et al*., 2013). It has been suggested that at up to a threshold of a few gigapascals, the survival rate is of order 1–10%, but that this rapidly falls off above that (Burchell, 2007) as increasing damage occurs to the structure of the cell and induces cell wall delamination as the shock wave passes through (Willis *et al*., 2006).

Impact testing on seeds, millimeter-scale objects, shows that they suffer internal damage and failure, when impact speeds reach around 1 km s^−1^, or typically ∼1 GPa (Jerling *et al*., 2008; Leighs *et al*., 2012). This suggests that larger, more complex bodies (compared to small, simpler ones) are at higher risk of internal damage caused by the passage of a shock wave. It is interesting to consider what happens to animals as well as seeds under these conditions. Given the current interest in tardigrades as organisms that can survive raw expose to space for at least short periods, their resistance to shock pressures may be the limiting factor in their success or otherwise as vectors for panspermia. The ability of tardigrades to survive extreme conditions is linked to their ability to enter a “tun” state in which they dehydrate, expelling 90%+ of their water, and produce antioxidants, which allows their metabolic rate to fall to 0.01% of normal. It is in this tun state that they were tested in the present study.

While the survival of tardigrades has been tested in static loading up to 600 MPa (Vasanthan *et al*., 2004) and 7.5 GPa (Ono *et al*., 2008), there is no knowledge of how they survive impact shocks. For bacteria, there is evidence that static and shock loads of the same magnitude have a significantly different effect on survival rates (Hazael *et al*., 2017). Accordingly, we have fired tardigrades at high speed in a gun onto sand targets, subjecting them to impact shocks and evaluating their survival.

## 2. Method

The animals used herein were the tardigrade species *Hypsibius dujardini,* which were handled according to the ethical rules for invertebrates with the consent of the departmental ethics officer. The tardigrades were fed mineral water and moss (see Fig. 1a, 1b). They were fired from a two-stage light gas gun (Burchell *et al*., 1999; Hibbert *et al*., 2017) at sand targets in a vacuum chamber. Prior to shooting, two or three tardigrades were loaded into a water-filled shaft in a nylon sabot (the number was measured in each case). The sabot was then frozen for 48 h so that the tardigrades were in a tun state during the shot. The sabot was then placed in the gun and fired at normal incidence into the sand. The whole sabot impacted the target in each shot. Impact speeds were measured in each shot to better than ±1% using two laser light stations mounted transverse to the direction of flight and focused onto photodiodes. The signals from the photodiodes, combined with their known separation (499 mm), provided the speed.

Six shots were executed at speeds from 0.556 to 1.00 km s^−1^ (see Table 1 for details of each shot). After each shot, the sand target was poured into a water column to separate the sand from other materials and isolate the tardigrades. The recovered tardigrades were then observed over time to discern whether they returned to a mobile state (*i.e.,* an active state). The time to achieve this was noted. As a control, tests were made to freeze 20 tardigrades and then defrost them without their being fired with the gun. All 20 were revived successfully, and it took them 8–9 h to recover to a mobile state, with none requiring more than 9 h. In an earlier study (Pasini *et al.,* 2014), tardigrades were frozen in an ice target that was impacted. Survival of the tardigrades in the target after impact was then evaluated. However, in that study, even in un-impacted frozen control samples, about two-thirds of the tardigrades died. The current study thus represents an improvement in the overall handling of the samples (with 20 out of 20 control samples surviving). The experimental method also provides a more uniform shock to the samples during the experiments, as a result of their being mounted in the small interior volume of the sabot, rather than their being distributed throughout the target.

**Table 1.** Impact Speeds per Shot and Peak Pressures for Water Ice Impacting Sand Calculated Using the Planar Impact Approximation

Impact speed (km s^−1^)	Peak shock pressure (GPa)
0.556	0.61
0.695	0.81
0.728	0.86
0.825	1.01
0.901	1.14
1.00	1.31

The peak shock pressure in each impact was estimated by using the Planar Impact Approximation (PIA) (Melosh, 2013). This requires a linear wave speed relationship for each material in the impact, which we simulate here as water ice impacting sand using the relevant coefficients from Melosh (2013) (see Table 1 for the calculated shock pressures and Table 2 for values of the relevant coefficients). When, as indicated in the discussion section, impacts on metals were modeled by using the PIA, the necessary coefficients were obtained from those of Ahrens and Johnson (1995) and are again given in Table 2.

**Table 2.** Coefficients Used for the Materials in the Planar Impact Approximation, which Uses a Linear Wave Speed Equation *U* = *C* + *Su,* Where *U* is the Shock Velocity and *u* is the Particle Velocity

Material	Density (kg m^−3^)	C (km s^−1^)	S	Reference
Ice	915	1.317	1.526	Melosh, 2013
Sand	1600	1.70	1.31	Melosh, 2013
Aluminum	2750	5.30	1.37	Melosh, 2013
Indium	7281	2.54	1.49	Ahrens and Johnson, 1995
Copper	8931	3.982	1.460	Ahrens and Johnson, 1995
Silver	10490	3.23	1.59	Ahrens and Johnson, 1995
Gold	19263	2.95	1.81	Ahrens and Johnson, 1995

## 3. Results

**FIG. 2.**
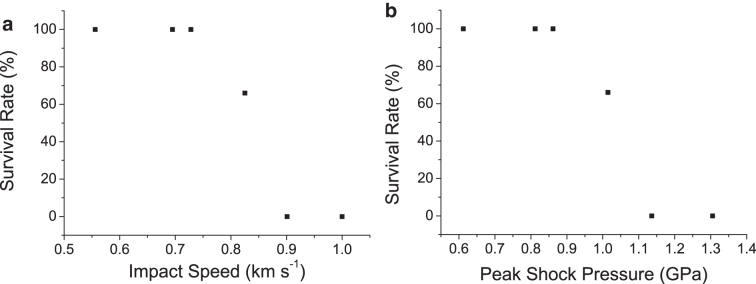
Results of impact experiments onto sand. (**a**) Tardigrade survival rate vs. impact speed. (**b**) Tardigrade survival rate vs. peak shock pressure.

The survival rate from each shot is shown in Fig. 2a versus impact speed and in Fig. 2b versus peak shock pressure. It can be seen that survival fell from 100% to 0% between 0.728 and 0.901 km s^−1^ (corresponding to 0.86–1.14 GPa). In the shots up to and including 0.825 km s^−1^, intact tardigrades were recovered post shot (*e.g.,* Fig. 1c), but in the higher-speed shots only fragments of tardigrades were recovered (*e.g.,* Fig 1d). Thus, shortly after the onset of lethality, the tardigrades were also physically broken apart as impact speed increased.

**FIG. 3.**
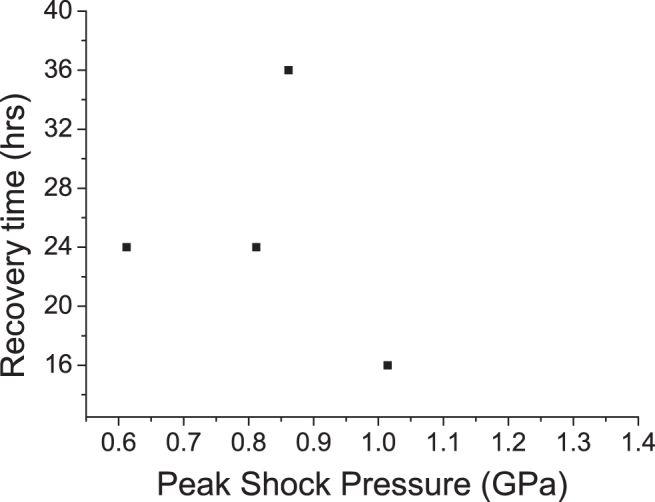
Recovery time post shot for those tardigrades which survived to regain mobility. All recovery times greatly exceed the 8–9 h recovery time from just being frozen.

For active tardigrades that were found post shot, Fig. 3 shows the recovery time to achieve a fully mobile state; these times were significantly greater than those for tardigrades in the frozen/defrosted control samples (8–9 h), which suggests that the impact shock had a more significant effect than freezing alone.

## 4. Discussion

Given that the results here suggest that peak shock pressures above 1.14 GPa will kill tardigrades, then it is likely that arrival of a tardigrade on Earth, for example by way of a meteorite impact, is not likely to be a viable means of a successful transfer even for such hardy organisms. There are other places in the Solar System, however, where biological material, during transfer, would encounter low shock pressures, which is discussed below. We also address the possible collection of sample material by spacecraft from the plumes of Europa and Enceladus.

### 4.1. Terrestrial ejecta impacting the Moon

It has long been proposed that ejecta (and fossilized material within) from giant impacts on Earth could have struck the Moon and become preserved (Armstrong *et al*., 2002; Armstrong, 2010; Burchell *et al*., 2014, 2017). Armstrong (2010) determined an average lunar impact speed for terrestrial ejecta of some 2.5 km s^−1^. However, it is the vertical component of impact speed that determines peak shock pressure; Figure 6 from the work of Armstrong (2010) shows that this has a mean value of about 1.3 km s^−1^. At vertical impact speeds of even 1 km s^−1^, the peak shock pressure in lunar impacts was estimated by Armstrong (2010) to be 2 GPa, *i.e.*, above that for tardigrade survival. Separately, the peak shock pressures for terrestrial material impacting the Moon have been calculated for a range of possible impactor and lunar surface material combinations, and shock pressures were found to be in the range of 2–5 GPa (Burchell *et al.,* 2017), which is consistent with those of Armstrong (2010), who pointed out, nonetheless, that 43% of impacts of terrestrial ejecta onto the lunar surface would be at speeds below 1 km s^−1^. Indeed, 29% of such impacts have a vertical impact speed of less than 0.5 km s^−1^, and 10% at less than 0.1 km s^−1^. These correspond to peak pressures of 0.5 and 0.02 GPa, respectively, which is well within tardigrade survival limits. Two qualifications, however, are required: (1) the degree of shock during ejection from Earth is also important, as is (2) the increase in temperature due to the shock impact. These points are discussed, for example, in the work of Halim *et al*. (2021); however, their simulations still show biomarkers potentially surviving in terrestrial ejecta that impact the Moon.

### 4.2. Martian ejecta impacting Phobos

As is the case for the Moon, a similar scenario can be applied to Mars, that is, martian impact ejecta striking Phobos. Chappaz *et al*. (2013) estimated that as much as 50 mg of martian surface material lies within every 100 g of phobosian regolith, of which 0.2 mg will have been deposited in the last 10 million years. Similarly, Ramsley and Head (2013) estimated that, over the past 500 Myr, around 250 ppm of martian surface material has been deposited in the phobosian regolith, and there is of order 6.5 × 10^8^ kg of martian ejecta in the phobosian upper surface. The impact speed on Phobos is estimated to range from 1 to 4.5 km s^−1^ (Chappaz *et al*., 2013), which, if typical material parameters are assumed, is likely to produce peak shock pressures just above those that permit tardigrade survival. However, even in the event some of this material was lightly enough shocked to permit tardigrade survival, long-term exposure to solar and cosmic radiation would still have sterilized much of it (Kurosawa *et al*., 2019).

### 4.3. Collection of material from the plumes of Europa and Enceladus

Active ejections of material into space is known to occur on some of the icy satellites of Jupiter and Saturn. Europa and Enceladus are known to have subsurface oceans (see Shematovich, 2018, or Hendrix *et al.,* 2019, for reviews of the ocean worlds), which vent into space via surface cracks, producing plumes (Spencer *et al*., 2006; Roth *et al*., 2014). These plumes have been sampled at Enceladus by the Cassini spacecraft, and both low- and high-mass organics have been observed (Postberg *et al*., 2018; Khawaja *et al*., 2019). The encounter speed with a space vehicle during a flyby is high; Cassini data was obtained at encounter speeds above 5 km s^−1^. Lower speeds in the 3.5–4.5 km s^−1^ range are possible if the saturnian orbit is optimized (Tsou *et al*., 2012), but this will still result in shock pressures on solid collectors of significantly greater than 1 GPa. However, if, as suggested by Tsou *et al*. (2012), a porous material such as aerogel were used as the collector (*e.g.,* see Burchell *et al*., 2006, for a discussion of the use of aerogel as a capture material in space), peak shock pressures have been calculated to be less than 1 GPa even at speeds of 6 km s^−1^ (Trigo-Rodríguez *et al*., 2008). At 5 or 6 km s^−1^, for impactors of 100 μm diameter and density 2500 kg m^−3^, approximately 1.5 cm thickness of aerogel would contain an impact for aerogel of density 50 kg m^−3^ (Burchell *et al*., 2009). This, however, rises to 15 cm depth of aerogel for a 1 mm impactor. The required aerogel depth would be decreased if the impact speed were lowered but would increase again for lower aerogel densities. These depths of aerogel, however, are not implausible and would permit capture of material at shock pressures below those that kill tardigrades.

**FIG. 4.**
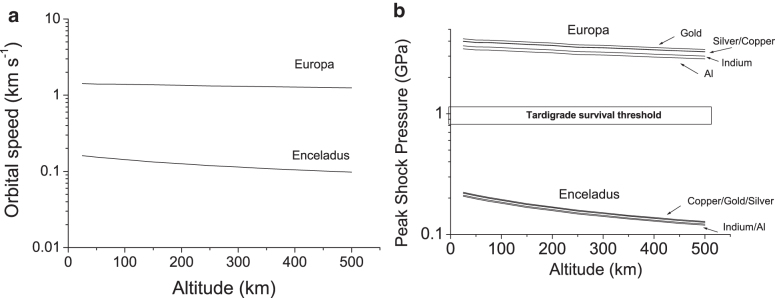
(**a**) Orbital speed vs. altitude at Europa and Enceladus. (**b**) Peak shock pressure on various metals as a function of altitude (note that within the resolution of the graphs the curves for several metals overlap). For Europa, all impacts produce pressures above the tardigrade survival limit, whereas for Enceladus impacts on all the metal surfaces are below the limit.

As an alternative to a flyby of an icy satellite, an orbiter could be employed. If the encounter is determined by the spacecraft orbital speed (and any contribution from the motion of the material in the plume is ignored), then the impact speed depends on the altitude of the orbit. These speeds can readily be predicted at both Europa and Enceladus (Fig. 4a). If we assume a range of metal collectors (as suggested by New *et al*., 2020, for collecting organic particles during a transit of Enceladus's plume), the peak impact shock pressures can be found by using the PIA. We have modeled aluminum, indium, copper, gold, and silver, as suggested in the work of New *et al*. (2020), with the necessary linear wave speed coefficients. The resulting shock pressures versus orbital altitude are shown in Fig. 4b (see also Tables 3 and 4). These values are well within survival limits for tardigrades at Enceladus, but they are too great for survival at Europa. Indeed, at the higher altitudes at Enceladus (and lower impact speeds), the main problem may be rebound of the impactor rather than the impactor sticking to the target, in which case a funnel-like arrangement may be needed to direct rebounding grains into a detector. We can thus envisage that, in a plume at Enceladus or Europa, a flyby mission could feasibly collect viable small animals such as tardigrades if an aerogel collector was used, and at Enceladus an orbiter could successfully use a solid metal target as well.

**Table 3.** Peak Shock Pressures Calculated in Enceladean Orbits for Water Ice Impacting Various Materials

Altitude (km)	Orbital speed (km s^−1^)	Peak shock pressure on aluminum (GPa)	Peak shock pressure on indium (GPa)	Peak shock pressure on copper (GPa)	Peak shock pressure on silver (GPa)	Peak shock pressure on gold (GPa)
25	0.161	0.207	0.212	0.220	0.220	0.224
50	0.154	0.197	0.201	0.209	0.209	0.213
75	0.148	0.188	0.192	0.200	0.199	0.203
100	0.143	0.181	0.185	0.192	0.192	0.195
150	0.133	0.167	0.171	0.177	0.177	0.180
200	0.126	0.157	0.161	0.167	0.166	0.169
250	0.119	0.148	0.151	0.156	0.156	0.159
300	0.114	0.141	0.144	0.149	0.149	0.151
350	0.109	0.134	0.137	0.142	0.142	0.144
400	0.105	0.129	0.131	0.136	0.136	0.138
450	0.101	0.123	0.126	0.131	0.130	0.132
500	0.098	0.120	0.122	0.126	0.126	0.128

**Table 4.** Peak Shock Pressures Calculated in Europan Orbits for Water Ice Impacting Various Materials

Altitude (km)	Orbital speed (km s^−1^)	Peak shock pressure on aluminum (GPa)	Peak shock pressure on indium (GPa)	Peak shock pressure on copper (GPa)	Peak shock pressure on silver (GPa)	Peak shock pressure on gold (GPa)
25	1.421	3.45	3.65	3.99	3.97	4.17
50	1.410	3.37	3.57	3.90	3.89	4.08
75	1.399	3.37	3.57	3.90	3.88	4.07
100	1.389	3.33	3.53	3.85	3.84	4.03
150	1.368	3.26	3.45	3.76	3.75	3.93
200	1.349	3.19	3.38	3.68	3.67	3.85
250	1.320	3.09	3.27	3.65	3.55	3.72
300	1.312	3.07	3.24	3.53	3.52	3.69
350	1.295	3.01	3.18	3.46	3.45	3.61
400	1.278	2.95	3.12	3.39	3.38	3.54
450	1.262	2.90	3.06	3.33	3.31	3.47
500	1.247	2.85	3.00	3.27	3.25	3.41

Collection of material from plumes also depends on the content of the plume and surface area of the collector. For example, it is estimated that the amount of material ejected in the Enceladus plume is 150–300 kg s^−1^, which, at a height of 80 km, produces 1 ice particle per m^3^ (Tsou *et al*., 2012). This suggests that an orbiter, as opposed to a flypast, would yield more samples. Further, the method by which material is entrained into the plume may not be straightforward, and the height to which larger (100 μm to millimeter scale) objects are ejected could be limited. Nevertheless, it does appear that, if larger objects could get into a plume, then animals such as tardigrades could survive capture by a passing spacecraft. Whether such a spacecraft should perform an analysis *in situ* or conduct a sample return to Earth is an open question. As noted in the work of Tsou *et al*. (2012), the lack of sample material and low sensitivity of *in situ* analysis tools would militate against *in situ* analysis. However, the cost of implementing planetary protection protocols for a sample return mission to a place potentially harboring life would dwarf the purely spacecraft-related mission costs.

## 5. Conclusions

We have shown that tardigrades can survive low- to moderate-speed impacts and the involved shock pressures at speeds up to 0.728 km s^−1^, but then survival was not observed at 0.901 km s^−1^ (corresponding to peak shock pressures of 0.86 and 1.14 GPa, respectively). The statistics involved are low, and future experiments with larger numbers would be beneficial. Future experiments should also assess what happens to the tardigrades below the survival limit. Tardigrades under these circumstances took significantly longer than the control samples to recover, which suggests that a degree of internal damage has to be overcome. Furthermore, it is not clear whether the reproduction cycle can be undertaken by the survivors. This was not observed after any shot in the present study, but the sample numbers were small and the samples kept isolated; thus further study is needed. Similarly, collecting samples of tardigrade eggs, using them in the projectiles, and then assessing whether they can develop afterward would also be a fruitful area of study.

That complex structures undergo damage in shock events is not a surprise. Willis *et al.* (2006) showed that, even for the simplest of cells, cell wall delamination was a factor in lethality. Further, the results of Jerling *et al*. (2008) and Leighs *et al*. (2012) suggest that larger organisms such as seeds also suffered internal damage due to passage of shock waves. That the tardigrades studied here sustained internal damage that resulted in lethality at a similar shock pressure to seeds is therefore not a great surprise. However, it is clear that shock also causes internal damage at lower shock pressures, as indicated by the longer recovery time required to restore mobility for the shocked specimens in comparison to those that were simply frozen and revived directly from the tun state. The peculiarity here may be that recovery and survival is still possible until just before the impact events begin to break the tardigrades apart.

When considering the implications for successful transfer of tardigrades (or similar organisms) across space, the low shock pressure required rules out most of the common scenarios of interplanetary transfer that involve impact speeds well above 1 km s^−1^ and shock pressures of many gigapascals. However, as indicated in the discussion section, there are niche environments where such transfers may be possible. These include transfer from planetary surfaces to nearby moons (*e.g.,* from Earth and Mars). Indeed, even when the average material involved is shocked above the survival limit, it may be possible that some experience a lesser shock, and survival may still occur. Similarly, if appropriate attention is given to the mission design (orbit or flyby) and collection method (solid collectors or underdense collectors such as aerogel), it may be possible to successfully sample the plumes of Europa and Enceladus for such life-forms. Indeed, the idea that these plumes may be responsible for icy satellite panspermia (*e.g.,* see Burchell *et al*., 2003, for a discussion) in their respective planetary systems could be investigated. Czechowski (2018), for example, considered enceladean ejecta and found that, although material can escape Enceladus under the correct conditions, it is unlikely to escape the saturnian system, leaving it available to impact other saturnian satellites. A successful transfer of viable material, of course, would depend on the impact speed on another satellite of the parent planet (with a limit of around 1 km s^−1^).

## Abbreviation Used

PIA: Planar Impact Approximation
